# A case report of multiple artery pseudoaneurysms associated with SARS-CoV-2

**DOI:** 10.3389/fcvm.2023.1174063

**Published:** 2023-05-19

**Authors:** Zixi Zhang, Yichao Xiao, Qiuzhen Lin, Chan Liu, Qiming Liu

**Affiliations:** ^1^ Department of Cardiovascular Medicine, The Second Xiangya Hospital, Central South University, Changsha, China; ^2^ Research Institute of Blood Lipid and Atherosclerosis, The Second Xiangya Hospital, Central South University, Changsha, China; ^3^ Modern Cardiovascular Disease Clinical Technology Research Center of Hunan Province, The Second Xiangya Hospital, Central South University, Changsha, China; ^4^ Cardiovascular Disease Research Center of Hunan Province, The Second Xiangya Hospital, Central South University, Changsha, China; ^5^ International Medical Department, The Second Xiangya Hospital, Central South University, Changsha, China

**Keywords:** SARS-CoV-2, pseudoaneursym, pulomary artery, gallbladder artery, case report, pathogensis

## Abstract

Arterial pseudoaneurysms are rare vascular abnormalities that can occur as a complication of infections. Artery pseudoaneurysms associated with SARS-CoV-2 are a rare occurrence in COVID-19 patients, and their rupture can result in significant hemorrhage and sudden death. Few cases of SARS-CoV-2-associated artery pseudoaneurysms have been reported, and their underlying pathophysiological mechanisms remain unclear. This study presents the first reported case of a patient who developed both pulmonary and gallbladder artery pseudoaneurysms following SARS-CoV-2 infection. We investigate the potential pathogenesis of these pseudoaneurysms and aim to improve the understanding of this rare complication.

## 1. Introduction

Severe acute respiratory syndrome coronavirus-2 (SARS-CoV-2) has been known to cause damage to organs and tissues containing angiotensin-converting enzyme 2 (ACE2) receptors, resulting in various complications (1). Direct damage to vascular endothelial cells can cause thrombosis, leading to pulmonary embolism or coronary heart disease (2). Although such events have been reported, reports on *de novo* pseudoaneurysms during coronavirus disease 2019 (COVID-19) infection are limited. This study presents a case of an 80-year-old male patient with chronic obstructive pulmonary disease (COPD) and type 2 diabetes mellitus who developed both pulmonary and gallbladder artery pseudoaneurysms one week after SARS-CoV-2 infection. The study aims to analyze the potential pathogenesis of these pseudoaneurysms to improve the understanding of this rare SARS-CoV-2-related complication.

## 2. Case report

**Figure 1:**
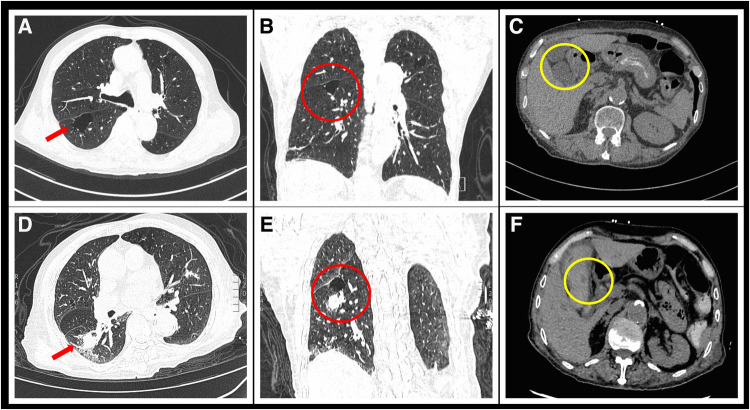
Comparison of chest and abdominal CT scans before and after admission. (**A**) Transverse section of chest HRCT upon admission shows a large bulla in the red arrow region due to COPD. (**B**) Coronal section image of the chest before hemoptysis shows a newly developed solid nodule in the right lung. (**C**) Abdominal CT at admission shows no abnormalities in the gallbladder artery. (**D**) Follow-up chest HRCT image 1 week after admission shows a new solid nodule with a diameter of 21 × 18 mm in the posterior segment of the right lower lobe (red arrow). (**E**) Coronal section image of the chest after hemoptysis shows a newly developed solid nodule in the right lung. (**F**) Emergency abdominal CT image taken after abdominal pain and vomiting shows a newly appeared circular low-density dark area at the gallbladder artery (yellow circle).

The patient experienced symptoms such as fatigue, muscle soreness, chills, fever, and unsteady gait three days prior to admission. Upon admission, laboratory tests showed significantly elevated levels of cardiac enzymes, transaminases, bilirubin, creatinine, erythrocyte sedimentation rate and procalcitonin. High-resolution computed tomography (HRCT) of the lungs showed multiple pulmonary cysts (Figure 1B), while abdominal CT revealed no significant abnormalities (Figure 1C). Sputum culture identified white Candida, but no bacteria or fungi were found in the bronchoalveolar lavage fluid. The initial diagnosis was acute myocarditis and liver and kidney failure following a novel coronavirus infection. Considering that the patient was positive for COVID-19, significantly elevated procalcitonin and inflammatory markers, paxlovid was given to inhibit viral replication, ceftriaxone and meropenem for infection, intravenous methylprednisolone succinate to reduce inflammation, and dibutyl cyclic adenosine calcium for injection to protect the heart and glutathione for injection to protect the liver. The patient had acute renal insufficiency, and his overall condition improved significantly after routine hemodialysis treatment.

**Figure 2:**
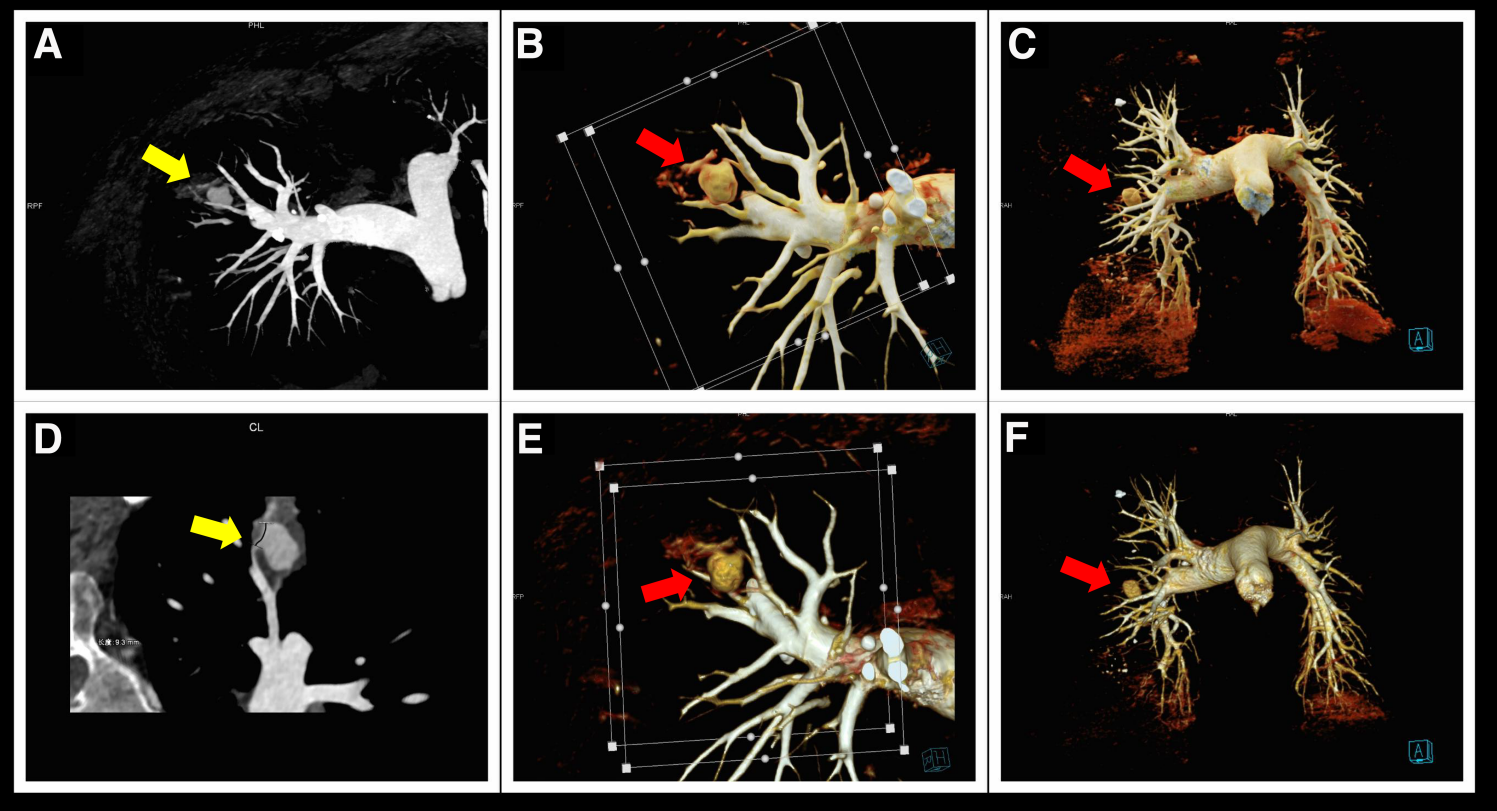
Contrast-enhanced CT scan of the patient's lungs. (**A,D**) Curved planar reconstruction images reveal the dilation of the pulmonary artery at its distal end, forming a pseudoaneurysm (yellow arrow). (**B,C,E,F**) Volume rendering technology images clearly show a nearly spherical mass pseudoaneurysm at the distal end of the right pulmonary artery (red arrow).

On the seventh day after admission, the patient experienced four sudden and unexpected episodes of hemoptysis, with each episode producing approximately 30–50 ml of blood. Hemostatic medication, i.e., etamsylate, hemocoagulase bothrops atrox and tranexamic acid, was administered immediately, and a repeat HRCT scan of the lungs revealed the presence of a newly formed solid nodule in the posterior segment of the right lower lobe (Figure 1D). Further contrast-enhanced CT imaging confirmed the diagnosis of a pseudoaneurysm of the pulmonary artery in the same location (Figure 2).

**Figure 3:**
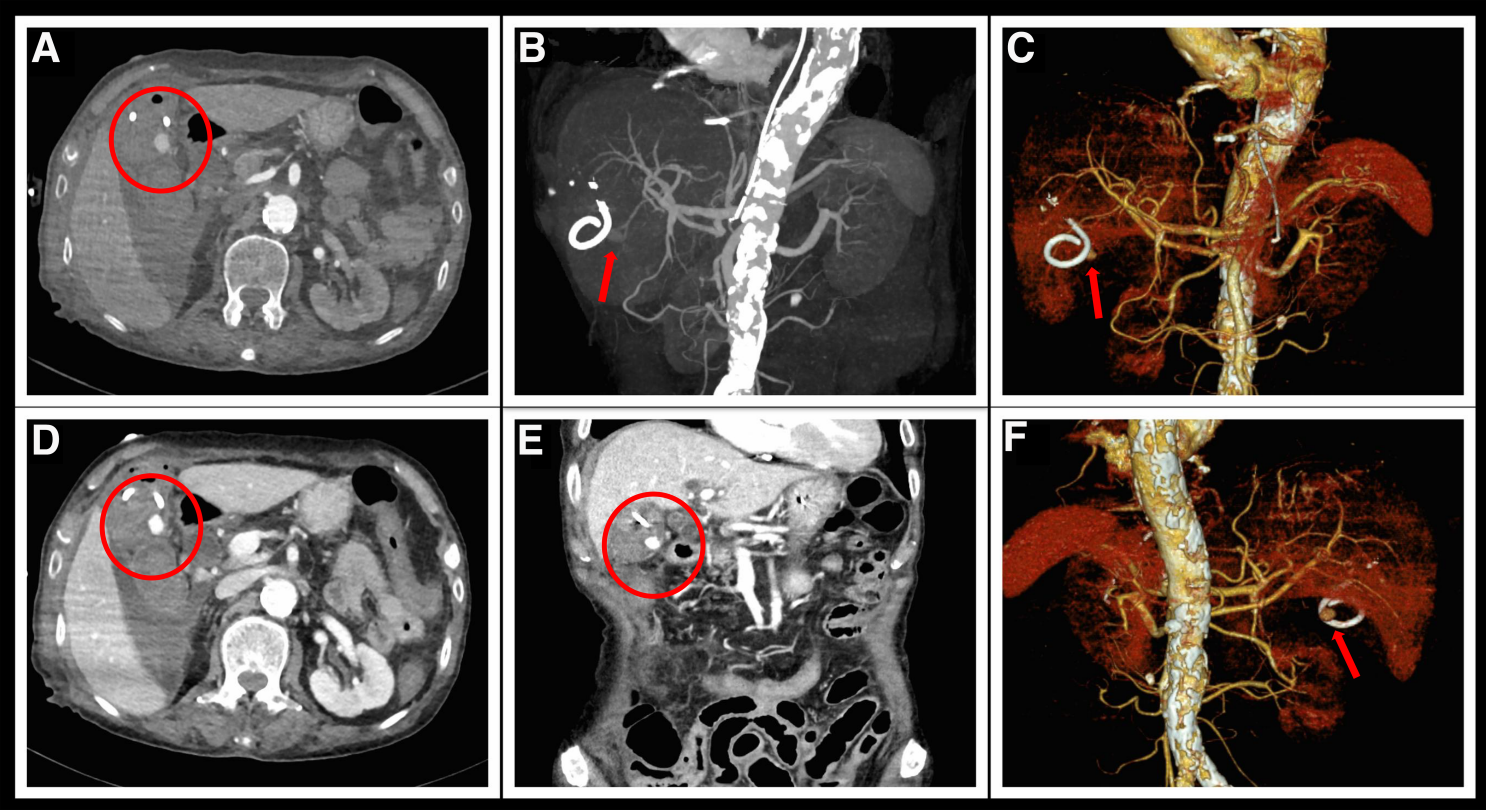
Aortic CTA examination after hematemesis. (**A,D**) Delayed enhancement of the pseudoaneurysm of the gallbladder artery (encircled in red) during the arterial phase of CTA. (**B,C,E,F**) Reconstructed three-dimensional images clearly show a spherical pseudoaneurysm at the exit of the gallbladder artery. (**E**) Reconstructed coronal section image shows an enhanced pseudoaneurysm image below the gallbladder.

After the cessation of hemoptysis, the patient developed severe right upper abdominal pain and recurrently vomited fresh blood. Emergency abdominal CT scan revealed acute cholecystitis with biliary bleeding (Figure 1F). With the assistance of interventional doctors, percutaneous cholecystocentesis and drainage were performed, resulting in the extraction of approximately 150 ml of bloody fluid. To identify the cause of abdominal pain and vomiting, we conducted computed tomography angiography (CTA) of the entire aorta, which revealed a newly formed pseudoaneurysm in the gallbladder artery (Figure 3).

A multidisciplinary consultation was organized to discuss further treatment options. Considering the patient's current physical condition, which was no longer suitable for surgical intervention, the experts recommended an interventional approach with balloon occlusion of the pseudoaneurysm. However, the patient subsequently experienced massive gastrointestinal bleeding with hemorrhagic shock, and due to the critical condition of the patient, he was transferred to the intensive care unit for further treatment. Ultimately, surgical intervention was abandoned in favor of conservative treatment.

## 3. Discussion

The formation of arterial pseudoaneurysms is characterized by the destruction of the arterial wall, leading to an extravascular hematoma surrounded by a thin layer of connective tissue. Pseudoaneurysms typically occur due to the focal dilation of arterial branches, and they are commonly associated with infections caused by pathogens like staphylococci, streptococci, or Mycobacterium tuberculosis (3). Although rare, arterial pseudoaneurysms have been reported as a complication of COVID-19, and their rupture can cause massive bleeding and hemorrhagic shock, which is a significant cause of death in patients with SARS-CoV-2 infection (Table 1).

**Table 1:** Pathophysiological mechanism of artery pseudoaneurysms associated with SARS-CoV-2.

Mechanism	Detail	Reference
Virus	Attack endothelial cells in blood vessels	(2)
Immune response	Multisystem inflammatory syndrome or “cytokine storm”, which can exacerbate vascular injury	(2)
Pulmonary cysts	Slow airflow within these cysts creates a conducive setting for the colonization of bacteria or fungi, which can worsen the intensity of pulmonary infections and inflammatory responses	(4)
Infection	Secondary mucormycosis infection	(5)
	The use of glucocorticoids during treatment can further suppress immune function, facilitating fungal invasion and pseudoaneurysm development	(6)
Complication	Diabetes is a chronic metabolic inflammatory disorder that activates serine kinases and leads to insulin resistance	(7)
	Prolonged diabetes and fluctuations in blood glucose can exacerbate endothelial damage and cardiovascular complications	(8)

The specific mechanisms that lead to the formation of pseudoaneurysms following SARS-CoV-2 infection remain unclear. However, existing evidence suggests that the virus can attack endothelial cells in blood vessels, and cause multisystem inflammatory syndrome, which can exacerbate vascular injury (2). The disruption of the endothelial layer can initiate the formation of pseudoaneurysms by increasing local wall stress and causing the tearing and rupture of the vessel wall. In some cases, severe inflammatory response to the virus, such as “cytokine storm”, may also induce the formation of the pseudoaneurysms. In addition, recent studies suggested that abnormal presence of autoantibodies and endothelial damage may also be cornerstones of COVID-19 disease (4, 9), but more clinical evidence is still needed.

Research indicates that patients recovering from SARS-CoV-2 infection may develop iatrogenic and noniatrogenic pulmonary cysts, which can rupture and merge to form lung bullae or spontaneous pneumothorax (5). Slow airflow within these cysts creates a conducive setting for the colonization of bacteria or fungi, which can worsen the intensity of pulmonary infections and inflammatory responses. This process is similar to the formation of cavities and pulmonary artery pseudoaneurysms after tuberculosis infection (6). In this case, the patient had a history of COPD and suboptimal baseline lung function. A large lung bulla is visible above the pulmonary artery pseudoaneurysm, and this structural anomaly could potentially contribute to the development of the pseudoaneurysm.

In addition, secondary mucormycosis infection following SARS-CoV-2 infection may also be an important mechanism for the formation of pseudoaneurysms (7, 8). Although there are few reports of this disease, it is plausible that SARS-CoV-2 infection can impair immune system, while the use of glucocorticoids during treatment can further suppress immune function, facilitating fungal invasion and pseudoaneurysm development. Notably, this condition appears to be more prevalent in elderly diabetic patients (10), as diabetes is a chronic metabolic inflammatory disorder that activates serine kinases and leads to insulin resistance (11). Prolonged diabetes and fluctuations in blood glucose can exacerbate endothelial damage and cardiovascular complications (12), increasing the risk of developing pulmonary artery pseudoaneurysms.

Although there have been no reports of SARS-CoV-2-related pseudoaneurysms in the gallbladder artery, the virus can damage organs that have ACE2 receptor (1), which may include the gallbladder artery, increasing the risk of pseudoaneurysm formation. If a patient presents with pseudoaneurysms in multiple locations, Behçet's disease, a chronic autoimmune disease characterized by vasculitis should be considered. However, in the case presented, the patient had developed pseudoaneurysms in both the pulmonary and gallbladder arteries but had no oral or genital ulcers, and the antibodies associated with rheumatic immunity were negative, ruling out Behcet's disease. Instead, the patient was diagnosed with multiple pseudoaneurysms that were likely related to SARS-CoV-2 infection, given the explosive inflammatory response observed and the rapid emergence of these pseudoaneurysms.

## 4. Conclusion

In conclusion, we present a case of pulmonary artery and gallbladder artery pseudoaneurysms that developed during SARS-CoV-2 infection and investigate the underlying mechanism of SARS-CoV-2-associated artery pseudoaneurysms. Elderly diabetic patients experiencing sudden hemoptysis or hematemesis during the recovery phase following SARS-CoV-2 infection should be evaluated for rare complications such as artery pseudoaneurysms. Early diagnosis and surgical intervention can significantly improve the long-term prognosis of patients with this condition.

## Data Availability

The original contributions presented in the study are included in the article, further inquiries can be directed to the corresponding author/s.
